# Cell Fate Potential of NG2 Progenitors

**DOI:** 10.1038/s41598-020-66753-9

**Published:** 2020-06-18

**Authors:** Rebeca Sánchez-González, Ana Bribián, Laura López-Mascaraque

**Affiliations:** Instituto Cajal-CSIC, 28002 Madrid, Spain

**Keywords:** Gliogenesis, Development of the nervous system, Cell fate and cell lineage, Glial progenitors, Neural progenitors

## Abstract

Determining the origin of different glial subtypes is crucial to understand glial heterogeneity, and to enhance our knowledge of glial and progenitor cell behavior in embryos and adults. NG2-glia are homogenously distributed in a grid-like manner in both, gray and white matter of the adult brain. While some NG2-glia in the CNS are responsible for the generation of mature oligodendrocytes (OPCs), most of them do not differentiate and they can proliferate outside of adult neurogenic niches. Thus, NG2-glia constitute a heterogeneous population containing different subpopulations with distinct functions. We hypothesized that their diversity emerges from specific progenitors during development, as occurs with other glial cell subtypes. To specifically target NG2-pallial progenitors and to define the NG2-glia lineage, as well as the NG2-progenitor potential, we designed two new StarTrack strategies using the NG2 promoter. These approaches label NG2 expressing progenitor cells, permitting the cell fates of these NG2 progenitors to be tracked *in vivo*. StarTrack labelled cells producing different neural phenotypes in different regions depending on the age targeted, and the strategy selected. This specific genetic targeting of neural progenitors *in vivo* has provided new data on the heterogeneous pool of NG2 progenitors at both embryonic and postnatal ages.

## Introduction

NG2-glia are the main proliferative neural cells in the adult brain, representing about 5% of all the CNS cells^1–3^. These cells are distributed homogeneously throughout the brain^4^ and they are considered to be an independent population of glial cells, otherwise known as oligodendrocyte progenitor cells (OPCs) given that they can differentiate into oligodendrocytes during development, in the adult brain or after injury^5–7^. One significant feature of NG2-glia is that they lack gap junction coupling yet they maintain a high input resistance, and they developing voltage-dependent sodium and potassium currents. These cells express ionotropic glutamate and GABA receptors and strikingly, they form direct synapses with neurons^8,9^, raising the possibility that they may modulate the activity of neurons and the operation of neural networks.

During development, NG2-glia give rise to cells of the oligodendrocyte lineage and to a few astrocytes^10–12^. In addition, these cells generate mature oligodendrocytes, although most of them do not differentiate into oligodendrocytes in the adult CNS^13,14^, rather they remain in a “progenitor” state. Indeed, the role of this undifferentiated population in the mature CNS remains largely unknown^15^. What is known is that they increase in number following traumatic insults) and that they contribute to the formation of glial scars, suggesting they help limit neurodegeneration^1,16^. Nevertheless, it is not clear whether these features are common to all NG2-glia cells or if there are different subpopulations that fulfil specific functions.

Besides oligodendrocytes, NG2-glia have the potential to generate a variety of cell types like astrocytes and neurons, both *in vitro* and *in vivo*, and at either postnatal or adult stages^17–20^, although the latter is still not fully clear^3,5,11,21,22^. This diversity could reflect the potential heterogeneity within the NG2-glia population based on their localization or on developmental factors that condition their activity and progeny^23^. Heterogeneity has been reported between NG2-glia populations in the gray and white matter^11,16,21,24–26^.

Recently, the StarTrack clonal analysis method was used to demonstrate that even in the adult brain, one single NG2-glia can generate up to 400 cells^27^. In this work, we use two independent StarTrack strategies to evaluate the NG2-glia lineage and NG2-progenitor potential by labelling these progenitor cells and following their cell progeny *in vivo*. Therefore, the origin, heterogeneity and function of NG2-glia are questions of great importance that remained unknown. Here, using new StarTrack strategies, directed to target NG2-progenitors at different developmental ages, we provide some answers to the cell fate and therefore, to the heterogeneity of this population of cells.

## Results

### StarTrack strategies to target the NG2 lineage

**Figure 1:**
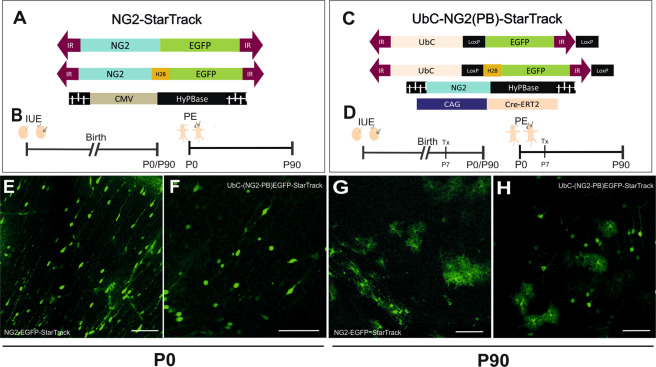
Experimental use of NG2 StarTrack constructs, and their short and long-term distributions. (**A**) Scheme of the *NG2-EGFP-StarTrack*constructs along with the CMV-HyPBase transposase. The *NG2-EGFP-StarTrack* contains inverted terminal repeats (IR) that the transposase recognizes, allowing it to randomly integrate copies of the NG2-StarTrack plasmids into the genome. **(B)** IUE was performed at E12, E14 or E16 and the animals were analyzed at short- (P0) and long-term (P90) intervals. PEs were performed at P0 and analyzed at P90. **(C)** The *UbC-(NG2PB)-EGFP-StarTrack* strategy involved using the *UbC-EGFP-StarTrack* plasmid with a NG2 promoter in the transposase and Cre-recombinase. **(D)** Embryos at E12, E14 or E16 and P0 pups were electroporated after ventricular injection of the StarTrack mixture. Tamoxifen was administered at around P7 in all the animals analyzed at P90. **(E)**Targeted pallial embryonic progenitors produced NG2-EGFP^+^ cells in the cortex with immature morphologies at P0, as well as different neural cell types at P90 **(G)**. **(F)** UbC-EGFP labelled cells were widespread throughout the cerebral cortex at P0 and P90 **(H)**. Scale bar 100 µm.

The StarTrack approach^28,29^ is based on the use of 12 plasmids that drive the expression of up to 6 different fluorophores in the tagged cells and their progeny after piggyBac-driven stochastic integration into the genome. This strategy was modified in order to target the NG2 lineage *in vivo* by using novel StarTrack plasmids carrying the NG2-promoter, *NG2-StarTrack*. We selected two StarTrack plasmids that drive EGFP expression in either the cytoplasm or the nucleus (fused to H2B histone: Fig. 1A). Since, we only used the plasmid encoding EGFP, we named this approach *NG2-EGFP-StarTrack*. The other plasmid was the HyPBase plasmid, with the hyperactive *PiggyBac* transposase under the control of the ubiquitous CMV promoter (Fig. 1), which recognizes the inverted terminal repeats (IRs). This allows to integrate the NG2-EGFP sequence directly into the genome of the transfected ventricular progenitors cells, independent of NG2-promoter activity, and enabling to track their NG2-cell progeny. Thus, after co-electroporation of the three plasmids, transfected cells in which the *NG2*-promoter is activated, will be tagged with EGFP, which can be detected in the brain when examined on postnatal days 0 (P0) or P90 (Fig. 1B).

To reveal the complete cell fate potential of the NG2-progenitor pool, irrespective of the lineage, the cytoplasmic and nuclear plasmids of the *UbC-StarTrack* were used, driven by a ubiquitous promoter and only encoding the gene encoding GFP. The hyperactive *PiggyBac* transposase was also modified to be driven by the NG2-promoter rather than the ubiquitous CMV promoter, referred to as *UbC-(NG2-PB)-StarTrack* (Fig. 1C). Targeting VZ progenitors with the *UbC-(NG2-PB)-EGFP-StarTrack* plasmid mix, allowed the entire cell progeny of active NG2-progenitors to be tracked independently of their lineage, even when the NG2 promoter is shut-off (Fig. 1D).

Both these strategies were used separately, targeting progenitors at different stages (E12, E14, E16 and P0), and analyzing following successful plasmid integration short- and long-term (Fig. 1E–H). At P0, EGFP^+^ cells were spread throughout the cortex, displaying an immature morphology (Fig. 1E,F). However, at adult stages labelled cells were seen in the pallial cortex and they displayed different neural morphologies, such as those of astrocytes, NG2-glia, oligodendrocytes and even neurons (Fig. 1G,H). Thus, *NG2-EGFP-StarTrack* strategy exclusively label the NG2 cell progeny. Conversely, NG2-hyPBase labelled only those progenitors with an active NG2-promoter, whereas all the different cell lineages generated by NG2 progenitors were labelled when the progenitors were targeted by *UbC-(NG2-PB)-StarTrack*.

### Targeting progenitor cells with the NG2 promoter

**Figure 2:**
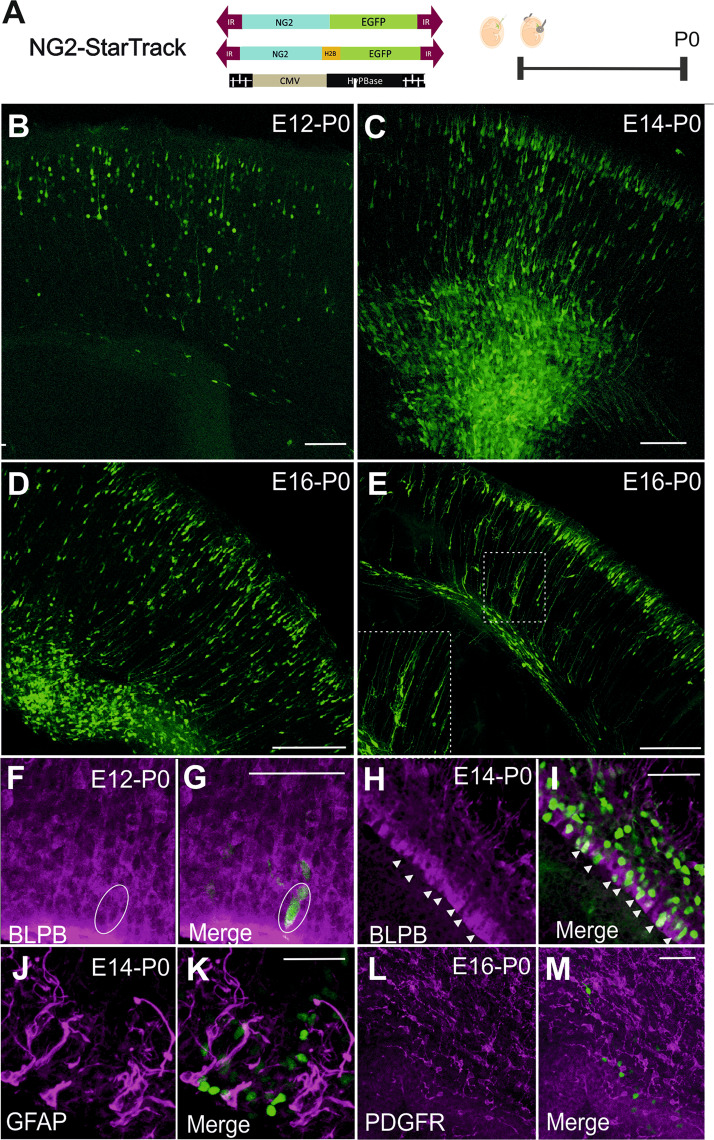
IUE of the NG2-EGFP-StarTrack at P0. **(A)** Scheme of IUE at different embryonic stages (E12-E16) using NG2-EGFP-ST with the CMV-HyPBase transposase. The tissue was analyzed at P0 in all cases. **(B)** Targeting progenitor cells at E12 produced EGFP^+^ cells at P0 with different immature morphologies stretching from the VZ to the cortical plate. **(C)** Targeted progenitors at E14 induced strong EGFP^+^ expression in NG2 cells widely distributed in pallial areas at P0. **(D)** After IUE at E16, EGFP^+^ cells were found at P0 close to the LV and in the upper layers of the cortical plate. **(E)** *NG2-EGFP-StarTrack* labelled cells displayed immature morphologies similar to radial glia-like cells, along with other immature morphologies (square). **(F**–**I)** BLBP labelling recognizes labelled radial glial cells at P0. **(J**–**M)** No co-localization with GFAP **(J)** and PDGFRα **(L)** was observed in NG2-EGFP^+^ cells close to the ventricle. Circles and head arrows display the coexpression of EGFP and BLBP positive cells. Scale bar 100 µm and 50 µm.

To selectively label NG2-progenitors and their progeny that express NG2, we used a modified StarTrack plasmid, *NG2-StarTrack*. After IUE of the *NG2-EGFP-StarTrack* mix into the dorsal VZ, a large number of EGFP^+^ cells could be seen throughout the cortex (Fig. 2A). At P0, immature EGFP^+^ cells targeted at E12 were found in several cortical layers, yet mostly within layer 3/4 (Fig. 2B). By contrast, those targeted at E14 and E16, were mostly situated in layers 2/3 (Fig. 2C,D). Remarkably, radial glia cells (RGCs) were evident close to the ventricle (Fig. 2E), as well as glial cells characterized by their bipolar morphology and branched processes (Fig. 2E, inset). In addition, many EGFP^+^ cells located close to the lateral ventricle wall expressed brain lipid binding protein (BLBP: Fig. 2F–I), a typical RGC marker. However, no co-localization was observed in NG2-EGFP+ cells close to the ventricle with GFAP (Fig. 2J,K) and PDGFRα (Fig. 2L,M), even some labelled cells located in cortical areas, outside the ventricle, were positive for PDGFR (data not shown). Thus, after targeting E12-E16 progenitors, the cells labelled at P0 were spread widely across the cortical plate, displaying immature morphologies. The dispersion of these cells was determined by the stage at which their progenitors were electroporated. In summary, *NG2-EGFP-StarTrack* allowed us to track immature cells that were spread widely across the cortical plate and that displayed spatio-temporal differences in their immature cell identity.

### Time course of the generation of specific NG2 cell progeny after targeting with *NG2-EGFP-StarTrack*

**Figure 3:**
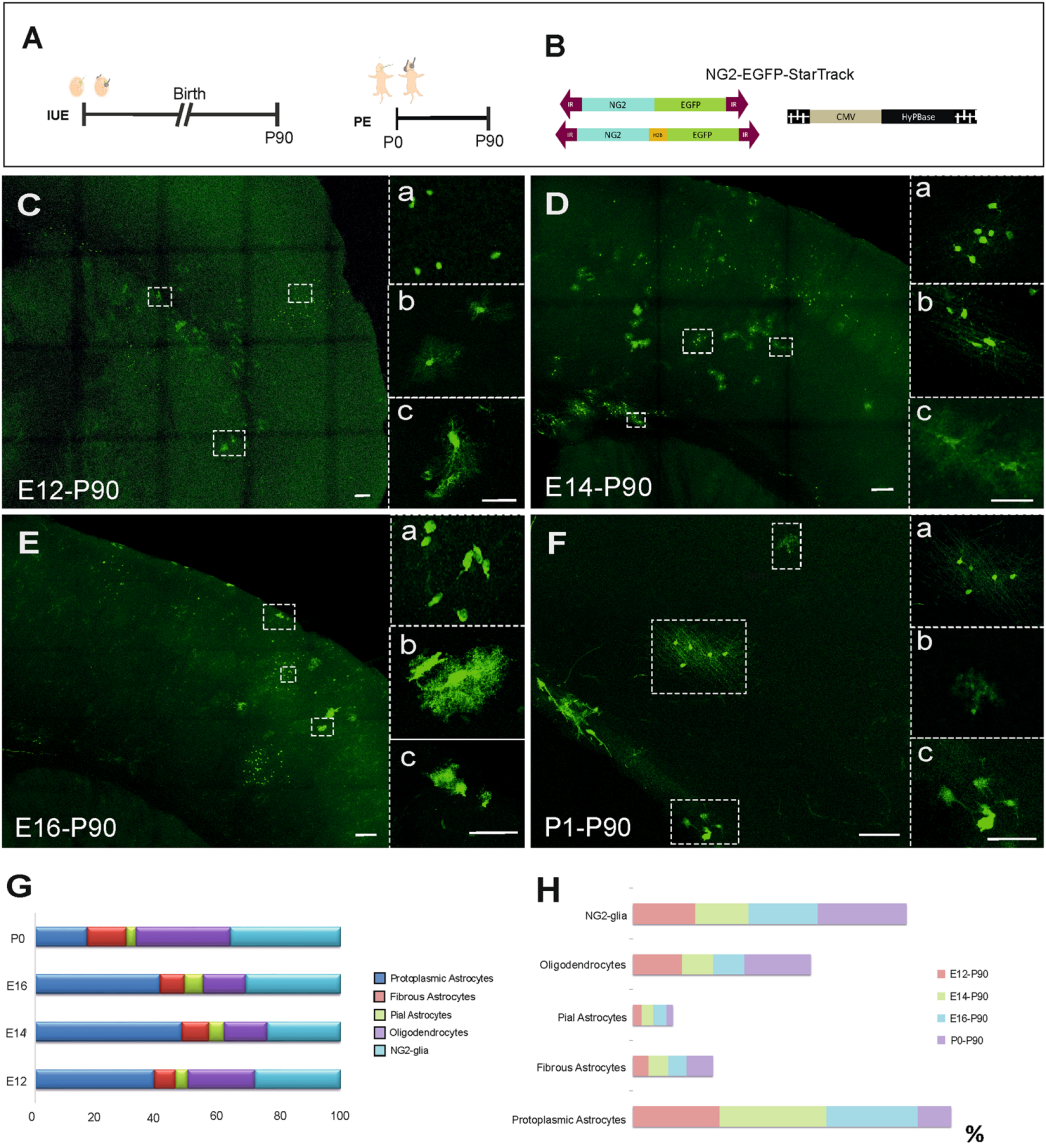
*NG2-EGFP-StarTrack* analysis in the adult brain. **(A)** Scheme of the *in vivo* experimental approach at different embryonic stages (E12, E14, E16) and P0. All brains were analyzed at P90 **(B)** representation of *NG2-EGFP-StarTrack* constructs along with the HyPBase. **(C)** EGFP^+^ cells in the cortex at P90 following IUE at E12 differentiated into: protoplasmic astrocytes (39%, b); and NG2 cells in the gray matter (28%, a); fibrous astrocytes in the Corpus Callosum (7%, c); and oligodendrocytes (22% of the total). **(D)** EGFP^+^ cells at P90 after E14 IUE develop into NG2-like cells (24%, a), oligodendrocytes (14%, b) and protoplasmic astrocytes (48%, c). **(E)** EGFP^+^ cells at P90 after IUE at E16 have developed into NG2-like cells (31%, a), astrocytes in the gray matter (41%, b) and pial astrocytes (6%, c), the largest number of differentiated cells after IUE. **(F)** Postnatal electroporation of the *NG2-EGFP-StarTrack* construct along with the CMV-HyPBase produced EGFP^+^ cells in the gray (a-b) and white matter (c). **(G)** The different proportion of neural cells depending the targeted progenitor cells (at least N = 3/age). **(H)** The graph shows the proportion of NG2, oligodendrocytes, protoplasmic-, fibrous- and pia-astrocytes at P90, from progenitor cells labelled at E12 (red), E14 (green), E16 (blue) or P0 (purple). Protoplasmic astrocytes are in blue, fibrous astrocytes in red, pial cells in green, oligodendrocytes in purple and NG2-cells in cyan. Slices 50 µm. Scale bar 100 µm and 50 µm.

To further characterize the efficacy of the new genomic tools in the adult brain, the *NG2-EGFP-StarTrack plasmid* was co-electroporated with the CMV-HyPBase at E12, E14, E16 and P0, to be analyzed at adult stages (P90, Fig. 3A,B). This strategy allowed the NG2-specific progeny of neural progenitors to be tracked independently of their mitotic activity. Electroporation of the *NG2-EFGP-StarTrack* plasmid into the dorsolateral part of the LV wall, produced a variety of cell types that were distributed throughout the cortical rostro-caudal axis (Fig. 3C–F). At P90, only a few embryonic tagged cells were identified as fibrous and pial astrocytes, as well as the oligodendrocytes predominantly located in the white matter (Fig. 3C–E). At P90, the percentage of NG2-glia increased as the cells were targeted later in development (E12, 24%; E14, 24%; E16, 31%; P0, 40%). Progenitor cells targeted at P0 gave rise to the NG2 lineage (70%), identified as oligodendrocytes (30%) and NG2-glia (40%), and the astroglial lineage (30%). At this respect, the percentage of astrocytes was relatively smaller compared to the results obtained from embryonic progenitors. Indeed, embryonic progenitors were mostly committed to the astroglial lineage (E12, 50%; E14, 61%; E16, 61%: Fig. 3G). In addition, some embryonic tagged cells pertained to the neuronal lineage, although their number decreased as development progressed (E12, 37%; E14, 23%; E16, 15%). However, after targeting postnatal progenitors (Fig. 3F,G), labelled cells were identified exclusively as glial cells since no neurons were found in the dorsal cortex (Fig. S1A). *NG2-EGFP-StarTrack* labelled cells were mostly committed to the glial lineage, astroglia and NG2-glia (Fig. 3H), although the distribution of EGFP^+^ glial cells differed significantly between the three embryonic progenitor subsets. NG2-glia and protoplasmic astrocytes were the most abundant EGFP^+^ cell types, more so than the pial and fibrous labeled astrocytes (Fig. 3H).

**Figure 4:**
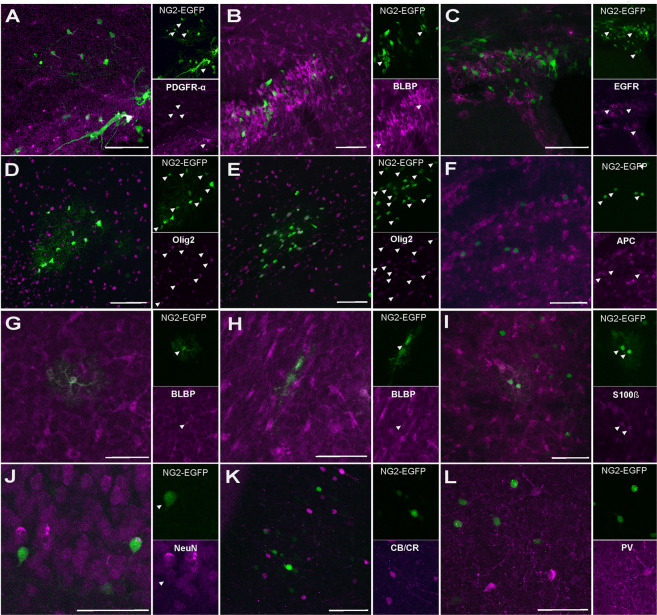
Identity of P90 labelled NG2 cells after targeting with *NG2-EGFP-StarTrack*. **(A)** Co-localization of PDGFR-α with *NG2-EGFP*- *StarTrack* labeled cells in the cerebral cortex and immature cells close to the wall of the lateral ventricle. **(B)** BLBP positive cells colocalized with *NG2-EGFP-StarTrack* cells close to the ventricle. **(C)** There are not colocalization with EGFR. **(D**,**E)** Cluster of NG2-like EGFP^+^ cells in the gray and white matter expressing Olig2. **(F)** APC was used to identify oligodendrocytes**. (G**,**H)** BLBP + cells colocalized with NG2-EGFP + protoplasmic and fibrous astrocytes **(I)** Also, EGFP^+^ protoplasmic astrocytes expressing S100-β. **(J)** NG2-StarTrack labelled cells expressing NeuN, but not CB/CR or PV immunomarkers **(K,L)**. Slices 50 µm. Scale bar: 50 µm.

**Figure 5:**
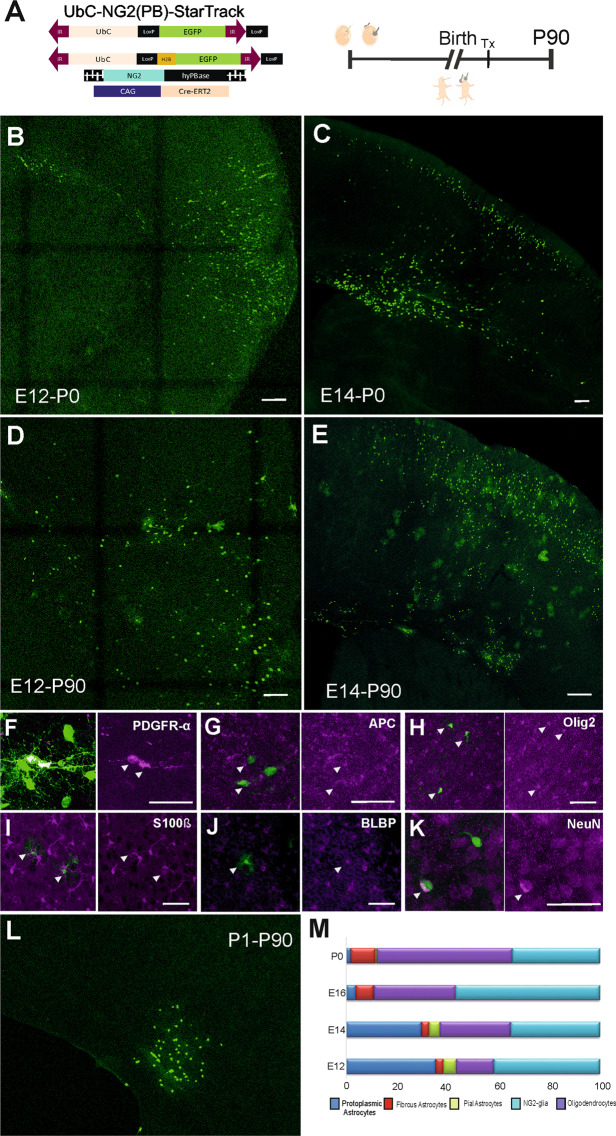
Short- and long-term tracking of NG2 transposase used in IUE/PE approaches. **(A)** Scheme of the *UbC-EGFP-StarTrack* plasmid, NG2-HyPBase transposase and Cre-recombinase. Electroporation was performed after ventricular injection of the StarTrack plasmids at E12, E14, E16 or P0. **(B,C)** EGFP^+^ cells at P0 showed typical immature morphologies in the cortical plate after E12 and E14 IUE, like RGCs or neuroblasts. **(D**–**L)** Long-term analyses of targeted cells revealed different neural cell types throughout the cortical white and gray matter: NG2-cells identified with PDFGRα **(F)**, APC **(G)** and  Olig 2**(H)**; astrocytes characterized using S100β **(I)** and BLBP **(J)** markers; and pyramidal neurons coexpressed NeuN **(K)**. **(L)** At P90, cells targeted at P0 produced cell clusters in the corpus callosum and fewer cortical cells. **(M)** The proportions of P90 labelled cells after targeting at E12, E14, E16 and P0 (at least N = 3/age). The total number of cells was obtained with the exclusion of neurons in all cases. The different cell types were defined as protoplasmic astrocytes (dark blue), fibrous astrocytes (red), pial astrocytes (green), NG2 cells (cyan) and oligodendrocytes (purple). The proportion of NG2-like cells was larger in E16 and P0 targeted cells. Slices 50 µm. Scale bar 100 µm and 50 µm.

The identity of the EGFP^+^ cells were first catalogued by morphology and location, and then by immunohistochemistry using specific markers for each lineage (Fig. 4). Immature cells close to lateral ventricle were characterized using PDGFRα (alpha-type platelet-derived growth factor receptor: Fig. 4A), BLBP (Fig. 2F–I; 4B) and EGFR (epidermal growth factor receptor: Fig. 4C). Both NG2-glia and astrocytes had an elongated morphology in the white matter and the classic stellate morphology in the grey matter. NG2-glia were identified by the expression of PDGFRα (Figs. 4A and 5F), while the Olig2 marker (oligodendrocyte transcription factor 2) identified both NG2-glia and immature oligodendroglia in gray (Figs. 4D and 5H) and white matter (Fig. 4E), and mature oligodendrocytes were distinguished by the expression of adenomatous polyposis Coli (APC/CC1: Figs. 4F and 5G). Astrocytes were identified using both BLBP in gray (Figs. 4G and 5J) and white matter (Fig. 4H) and S100β (calcium binding protein beta: Figs. 4I and 5I). While NeuN (neuronal nuclei) was used to label mature neurons (Figs. 4J, 5K and S1D). Calretinin/Calbindin (CR/CB) and parvalbumin (PV) were also used to characterize these neurons (Fig. 4K,L). There were not any colocalization with these markers, concluding that labelled neurons are not interneurons.

In conjunction, our data revealed that the neuronal/glial cell fate of neural progenitors changes with age. As such, embryonic progenitors of the ventricular zone were more committed to produce glial cells than neurons at E16 and P0 than at earlier stages (E12, E14), while postnatal progenitors almost exclusively give rise to glial progeny, mainly NG2 glia.

### Time course of the different cell lineages after targeting NG2-progenitors

To determine the fate of NG2 progenitor cells we performed electroporation of embryos with *NG2-EGFP-StarTrack*, producing EGFP^+^ cells that belong to both astrocytic and neuronal lineages (Fig. 2B–E). The reason that different cell lineages were labelled is that the NG2 promoter in progenitor cells remains active in mature cells. Thus, we designed a new strategy to specifically label NG2-expressing progenitors and their complete cell progeny by replacing the CMV-promoter of the HyPBase transposase with the NG2-promoter. The goal was to target those progenitor cells with an active NG2 promoter, defined as NG2 progenitors, using the transposase encoding NG2 promoter, and to maintain stable labelling in all their cell progeny irrespective of the cell lineage with the *UbC-StarTrack* construct. The *UbC-EGFP-StarTrack* plasmid and the new *NG2-HyPBase* (*UbC-(NG2-PB)-StarTrack*: Figs. 1B and 5A) construct were co-electroporated at embryonic (E12-E14) and postnatal stages (P0) to compare the EGFP^+^ cells labelled with the *NG2-StarTrack* at P0 (Fig. 5B,C) or in adults (P90, Figs. 5D,E,L). At P90, EGFP^+^ cells adopted neuronal morphologies in different cortical layers depending on the time of electroporation (E12 61%, E14 52%, E16 4%: Fig. S1B). In comparation with *NG2-EGFP-StarTrack* labeled neuronal cells there were none significant differences, but NG2-progenitor cells at E16 displayed lower neuronal fate potential. They presented the tipical pyramidal neuron morphology and coexpressed NeuN (Figs. 5K and S1D). Otherwise, there were more NG2 glial cells or oligodendrocytes than those found after *NG2-EGFP-StarTrack* electroporation (Fig. 5F–H). Futher, astrocytes were found in gray matter, white matter and pial surface and were charactericed with S100β and BLBP (Fig. 5I,J)

Using *UbC-(NG2-PB)-StarTrack* we assumed that the cells derived from NG2-progenitors were targeted at the time of electroporation. Further, NG2 expressing progenitors are active at different embryonic (E12, E14, E16) and postnatal ages (P0; Fig. 5L). However, at P0, the labelled cell clusters were smaller than those labelled by *NG2-EGFP-StarTrack*, suggesting that the progenitor cells could switch off or downregulate the NG2 promoter in the ventricular zone at early postnatal stages.

**Figure 6:**
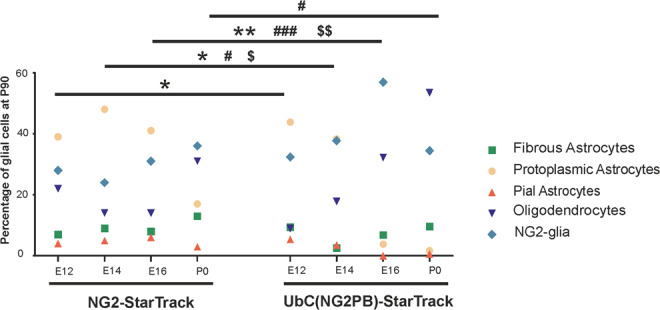
Comparative graph showing the proportion of glial cells at P90 after targeting progenitors at different developmental ages, using either *NG2-StarTrack* or *UbC-(NG2PB)-StarTrack*. The different glial cell types correspond to fibrous astrocytes (green), protoplasmic astrocytes (soft orange), pial astrocytes (orange), NG2 cells (blue) and oligodendrocytes (purple) per developmental age and strategy. Statistically significant differences across the NG2-glia are indicated by asterisks: *P  <  0.05, **P  <  0.01. Statistically significant differences across the protoplasmic astrocytes are indicated by pound sign: ^#^*P*  <  0.05, ^##^*P*  <  0.01, ^###^*P*  <  0.001. Statistically significant differences across the oligodendrocytes are indicated by dollar sign: ^$^*P*  <  0.05, ^$$^*P*  <  0.01.

Comparing temporal analysis of StarTrack approaches reveal that the adult cell progeny from E16-progenitors displayed the highest differences between the cell proportion of NG2-glia, protoplasmic astrocytes and oligodendrocytes depending of the strategy (Fig. 6). The percentage of glial-derived cell progeny (fibrous-, protoplasmic-, and pial-astrocytes; oligodendrocytes and NG2-glia), after targeting progenitor cells with *NG2-EGFP-StarTrack* (Fig. 3G) and *UbC- NG2(PB)-EGFP-StarTrack* approaches (Fig. 5M) at different developmental ages suggest that progenitors could undergo a temporal specialization, but it is necessary to target a precise population of NG2-progenitors to achieve their cell fate to later perform a specific clonal analysis of NG2-glia with the appropriate StarTrack approach.

In summary, here we provide new data on the adult NG2-cell progeny using the following *in vivo* approaches: (1) *StarTrack-NG2-EGFP* to track the NG2 cell progeny; and (2) *UbC-(NG2-PB)-StarTrack* to track the complete cell progeny of NG2-progenitors. Through both approaches, we found labelled cells belonging to neurogenic and gliogenic cell lineages, although their distribution and dispersion differed. NG2 progenitors targeted at E16 were mostly committed to become NG2-glia and oligodendrocytes, while E12 or E14 progenitors give rise to different neural types, mainly astrocytes but even neurons. It is remarkably that there were no neurons among the progeny if the cells were labelled at P0.

## Discussion

This study provides novel insights into the postnatal and adult cell fate of embryonic and postnatal NG2-cell progenitors. Using two new StarTrack strategies, we traced the entire postnatal and adult cell progeny of pallial NG2-progenitors *in vivo* from early stages of development. First we tracked the specific adult NG2 cell progeny using the *NG2-EGFP-StarTrack* and subsequently, using the* UbC-(NG2-PB)-EGFP-StarTrack* we tracked the complete NG2-derived progeny from progenitor cells with active NG2-promoter at the time the electroporation, regardless of their cell lineage. Both these approaches revealed the postnatal and adult fate of embryonic and postnatally targeted active NG2 cells, with NG2-glia associated with the oligodendrocyte lineage since they were discovered to be OPCs^9,24,28^. NG2-glia are thought to not only represent a transitional stage in the oligodendroglial lineage but also, a specific glial cell type with particular properties and functions^3,23^. However, their ability to differentiate into other cell types like astrocytes and neurons remains controversial^18,29–32^, particularly given the lack of experimental tools that target specific progenitors.

*In vitro* experiments showed that OPCs/NG2 could return to a multipotent state, suggesting that oligodendroglial lineage-restricted precursors might have a latent stem cell potential^14,33^. Using transgenic approaches to label NG2 cells, *in vivo* studies suggested these cells have the potential to become interneurons in both hippocampus and olfactory bulb^17,19^, neurons in the piriform cortex^31,34^ or astrocytes in the ventral areas of the brain and spinal cord^21,34^. Moreover, time lapse experiments demonstrated the multipotent potential of progenitor cells to give rise to interneurons or NG2 glia depending on their location in the SVZ^35^. Alternatively, genetic lineage-tracing experiments using Tx-inducible mice suggested that NG2-cells are restricted to the oligodendroglial lineage in adulthood^11,24,31,36,37^. Nevertheless, here using modifications of the StarTrack genetic tool, we tracked different cell progenies, including NG2-glia, oligodendrocytes, astrocytes and neurons, depending of the ventricular locations and developmental ages of the targeted ventricular NSCs. Further, this methodology avoids genetic mouse manipulation allowing variations in the promoter activity. Thus, while studies using Cre systems in NG2/PDGFRalpha cells showed just glial cell progenies, these new StarTrack approaches revealed that pallial E12 and E14 progenitor cells, with an active NG2 promoter, can give rise to neuronal cells in adults, as reported using transgenic approaches^17,18,31,34,35^ and even progenitor cells^38^. However, NG2 pallial progenitors, StarTrack-targeted from E16 onwards, are preferentially committed to the oligodendroglial lineage as previously reported^11,24,31,36,37^. These data suggest that NG2 progenitors are specified much earlier in the pallial ventricular zone. Thus, the number of pallial progenitors with an active NG2 promoter increased significantly from E12 to E16, in contrast to previous data from the fate mapping of Cre mouse strains^39^. Actually, our data also reveal that this number decreases in the postnatal NG2-progenitor cells, which could be related to the different waves of cortical NG2-glia generated from sub-pallial progenitors that migrate into the cortex at around E16^39^. Otherwise, NG2 and PDGFRα expression is rapidly down-regulated when NG2 cells start to differentiate into mature oligodendrocytes^40^.

In relation to their ventral origin of oligodendrocytes, it was suggested that the first OPCs/NG2-glia originating in ventral regions arrive in the pallium after E14^39^. However, we already proposed that pallial-derived NG2 clones probably increase their size at the expense of their direct differentiation of ventral-derived NG2 clones into oligodendrocytes. In fact, E14 pallial progenitors produced the largest NG2-cell clones derived from the neural tube^27^. Since we used a StarTrack system under the control of the hGFAP promoter in that earlier work^27^, we could not follow the complete cell differentiation process because this hGFAP promoter was not active in other glial and neuronal populations. Here, using the *UbC-(NG2-PB)-StarTrac*k, we show that embryonic NG2-progenitors sited in the pallium give rise to different glial cell types but also some neurons. In addition, NG2-progenitors also give rise to interneurons located at different layers of the adult olfactory bulb^41^.

In summary, optimizing StarTrack strategies appears to be a promising means to specifically assess the cell potential of NG2-progenitors that may be comprised of several pools of cells that generate the different cell lineages. These multipotent progenitors form a heterogenous pool that probably possess diverse functions. Thus, these data reinforce the idea that NG2-glia could behave as neural stem cells, suggesting potential therapeutic strategies for damaged CNS.

## Materials and Methods

### Animals

Wild type C57BL/6 mice were housed at the Cajal Institute animal facility. The day of vaginal plug detection was defined as embryonic day 0 (E0) and the day of birth as postnatal day 0 (P0). Depending on the experiment, the mouse’s brain was analyzed on the day of birth (P0) or three months later (P90: Table 1). All procedures for handling and sacrificing animals complied with all relevant ethical regulations for animal testing and research. All the experiments were performed in compliance with the European Commission guidelines for the welfare of experimental animals (2010/63/EU) and the Spanish Ministry of Agriculture (RD1201/2005 and L32/2007). All procedures were approved by the CSIC and the Community of Madrid Ethics Committees on Animal Experimentation in compliance with National and European legislation (PROEX 223/16). For all experiments were used at least n = 3.

**Table 1:** Time of electroporation and analysis of the *NG2-EGFP-StarTrack* and *UbC-(NG2PB)-EGFP-StarTrack* labelling.

Age of electroporation	Time point analyzed	
E12	P0	P90
E14	P0	P90
E16	P0	P90
P0	—	P90

### Tissue processing

Early postnatal mice (P0) were anesthetized by hypothermia and adult mice (P90) by intraperitoneal injection of Equithesin (3 mL/kg body weight). The mice were them perfused transcardially with 4% paraformaldehyde (PFA) in 0.1 M phosphate buffer (PB, pH 7.2). The brains were isolated and post-fixed in PFA for 2 hours at 4 °C and 50 µm coronal vibratome sections were obtained.

### Design of the *StarTrack* plasmids

*NG2-EGFP-StarTrack*: We modified the *StarTrack PiggyBac* PB-GFAP-EGFP (enhanced green fluorescent reporter protein) plasmid, under the control of the human GFAP (hGFAP) gene promoter^42,43^, to generate PB-NG2-EGFP containing the whole mouse NG2 promoter (mNG2 promoter) instead of the hGFAP promoter, in order to follow the potential of the progenitor cells with an active NG2-promoter. This mNG2-promoter was kindly provided by Dr Sellers, (University of Washington), who previously validate its transcriptomic activity^44^. The other plasmid encoded the hyperactive transposase of the PiggyBac system (HyPBase) under the control of the ubiquitous cytomegalovirus promoter (CMV) was kind gift from Prof. Bradley. Plasmids were generated by standard cloning methods and briefly, oligonucleotides (Sigma-Aldrich) and their PCR products were cloned using CloneJET PCR Cloning Kit (Fermentas) and cohesive restriction enzymes (Fermentas). Fragments were then ligated using the Fermentas Rapid DNA Ligation Kit (Fermentas) following the manufacturer’s instructions. The resulting products were transformed into E. coli JM107 bacteria using the “Transform Aid” Bacterial Transformation Kit (Fermentas). The vector containing the hyperactive transposase of the PiggyBac system, p-CMV-HyPBase (HyPBase), was kindly provided by Prof. Bradley.

*UbC-(NG2-PB)-StarTrack*: In this strategy we used the *UbC-EGFP-StarTrack* plasmid^45,46^ and a new transposase encoding the mNG2 promoter. For this purpose, the CMV promoter of the p-CMV-HyPBase plasmid was replaced with the murine NG2-promoter kindly provided by Dr Frank Kirchoff^12^. This strategy allowed us to follow the entire cell progeny of targeted NG2-progenitors irrespective of their lineage. This mixture contains also pCAG-CreER^T2^ vector, necessary to eliminate the episomal copies of the *UbC-StarTrack* plasmids in those system cells leaving the cell cycle soon after electroporation and can maintain construct carrying NG2-EGFP in the cytoplasm with no need of integration^45,46^, kindly provided by Connie Cepko (Addgene #14797). In proliferative cells, as glial cells, constructs that do not integrate will be diluted during respective cell cycle divisions.

### In utero electroporation (IUE)

IUE was performed as described previously^42,46^. In summary, dams carrying E12 to E16 mouse embryos were anesthetized by inhalation of 1.5% isofluorane/O_2_ and maintained at 37 °C throughout. An antibiotic (Baytril 5 mg/kg: Bayer) and anti-inflammatory/analgesic (Meloxicam 300 µg/kg: VITA Laboratories) were administrated subcutaneously prior to surgery. The dam’s uterine horns were exposed by midline laparotomy and intraventricular lateral injections of the plasmid mixture at E12 (0.5 µg/µL in distilled water with 0.2% of Fast Green) were guided by ultrasound (VeVo 770; VisualSonics), whereas from E14 onwards they were visualized using cold light trans-illumination. For electroporation, the positive electrode was positioned on the dorsal part of the injected lateral ventricle (LV) to target the pallial progenitors. Five consecutive square electric pulses of 50 ms were then delivered through the uterus at 950 ms intervals. The voltage pulse varied depending on the embryonic stage (Table 2), and the surgical incision was then closed and the embryos were allowed to develop until the ages selected (P0 and P90). Animals were monitored until recovery and given post-operative care for the following 3 days.

**Table 2:** Different voltage rates applied during IUE and PE depending on the embryonic or postnatal stage.

	E12	E14	E16	P1
Voltage (V)	28	33	37	100

### Postnatal electroporation (PE)

After anesthetizing P0/P1 pups by hypothermia, PE was performed as described previously^46,47^ using cold light trans-illumination to visualize the lateral ventricles. The ventricular cavity was injected with the plasmid mixture and five consecutive square electric pulses of 100 V of 50 ms were delivered thorough the head at 950 ms intervals. Electroconductive LEM Gel (DRV1800, Moretti S.P.A.) was placed on both electrode paddles to avoid damaging the pups and to achieve successful current flow. The positive electrode was positioned above the pallial wall of the ventricular zone (VZ), directing the negatively charged DNA to the progenitor cells in the pallial ventricular wall. After electroporation, the pups were allowed to recover on a thermal plate and they were then returned to their mother. The litter and the dams were monitored three days after surgery.

### Tamoxifen administration

Tamoxifen (Tx, Sigma-Aldrich) was dissolved in corn oil (Sigma-Aldrich) at 37 °C to a final concentration of 20 mg/ml. Tx was administered via intraperitoneal injection as a single dose at approximately 5 mg/kg body weight. Pregnant mice were injected one day after IUE for the short-term analysis, whereas the pups were injected one day after birth in the long-term experiments. The mice were analyzed at least 3 days after injection.

### Immunohistochemistry

The identity of EGFP-labelled cells was revealed using different markers (see Table 3). First, the tissue was permeabilized with phosphate buffer saline containing Triton-X 100 (PBS-T) first at 0.5% and then, at 0.1%, (three times in a row respectively). Coronal vibratome sections (50 μm thick) were incubated for at least 30 minutes in blocking solution (5% normal goat serum –NGS- in PBS-T 0.1%) and then overnight at 4 °C with the primary antibodies (each antibody dilution is indicated in Table 3). The following day, the sections were washed several times with PBS-T 0.1% and incubated for up to 2 h with Alexa far red goat anti-rabbit or goat anti-mouse IgG (1:1000, Alexa Fluor 633 or 647, Molecular Probes) depending on the nature of the primary antibody. Finally, the sections were washed in PBS-T 0.1% and the three more times with PBS alone. The sections were mounted with Mowiol (Polysciences, IncI) and examined on a Leica TCS-SP5 confocal microscope.

**Table 3:** List of primary antibodies used for the molecular characterization of the labelled cells in the adult cortex.

Antibody	Abbreviation	Use	Species	Source
Adenomatous polyposis coli	APC	1:300	Mouse	Calbiochem
Brain lipid binding Protein	BLBP	1:500	Rabbit	Millipore
Calbindin/Calretinin	CB/CR	1:500	Rabbit	Abcam
Glial fibrillary acidic protein	GFAP	1:1000	Rabbit	Dako
Epidermal growth factor receptor	EGFR	1:150	Rabbit	Millipore
Neuronal nuclei	NeuN	1:500	Mouse	Millipore
Oligodendrocyte transcription factor 2	Olig2	1:1000	Rabbit	Millipore
Alpha-type platelet-derived growth factor receptor	PDGFRα	1:1000	Rabbit	Cell Signaling
Parvalbumin	PV	1:500	Rabbit	Swant
S100 calcium binding protein beta	S100β	1:500	Rabbit	Abcam

### Image analysis and data processing

Fluorescent labeling was visualized under an epifluorescence microscope (Nikon, Eclipse E600) with EGFP (FF01-473/10) and Cy5 (FF02-628/40-25) filter cubes (Semrock). The images were then acquired on a Leica TCS-SP5 confocal microscope adjusting the settings to the excitation and emission conditions of the fluorophores. Maximum projection images were obtained using LASAF Leica software, and the set-up for threshold and background parameters was the same for all images. CorelDRAW Graphics Suite 2018 software was used to prepare the figures (Corel Corporation, Canada). The counter cell plugin of ImageJ was used to count cells. Cells pooled all the animals together with the exclusion of neurons in the total number of cells to obtain the cell proportion in Figs. 3 and 5.

Statistical analysis was carried out using two-tailed unpaired Student’s t-tests (SigmaPlot 13 software, SPSS Science, Inc.). Values required a confidence interval of 95% (p < 0.05) and critical values of *P < 0.05, **P < 0.01 and ***P < 0.001 were adopted to consider values statistically significant. Graphs were obtained with Microsoft Excel, CorelDraw or Prism 6 (GraphPad) software.

## Supplementary information

Supplementary Information.
