# Supplementary material for: Cell Fate Potential of NG2 Progenitors
Source: Sci Rep. 2020 Jun 18;10:9876. doi: 10.1038/s41598-020-66753-9 (PMC7303219; doi:10.1038/s41598-020-66753-9)
Supplement: Supplementary file 1 — Supplementary Information. [file 41598_2020_66753_MOESM1_ESM.pdf]

Supplementary material

CELL FATE POTENTIAL OF NG2 PROGENITORS

Rebeca Sánchez-González*, Ana Bribián*[1], Laura López-Mascaraque

Figure S1

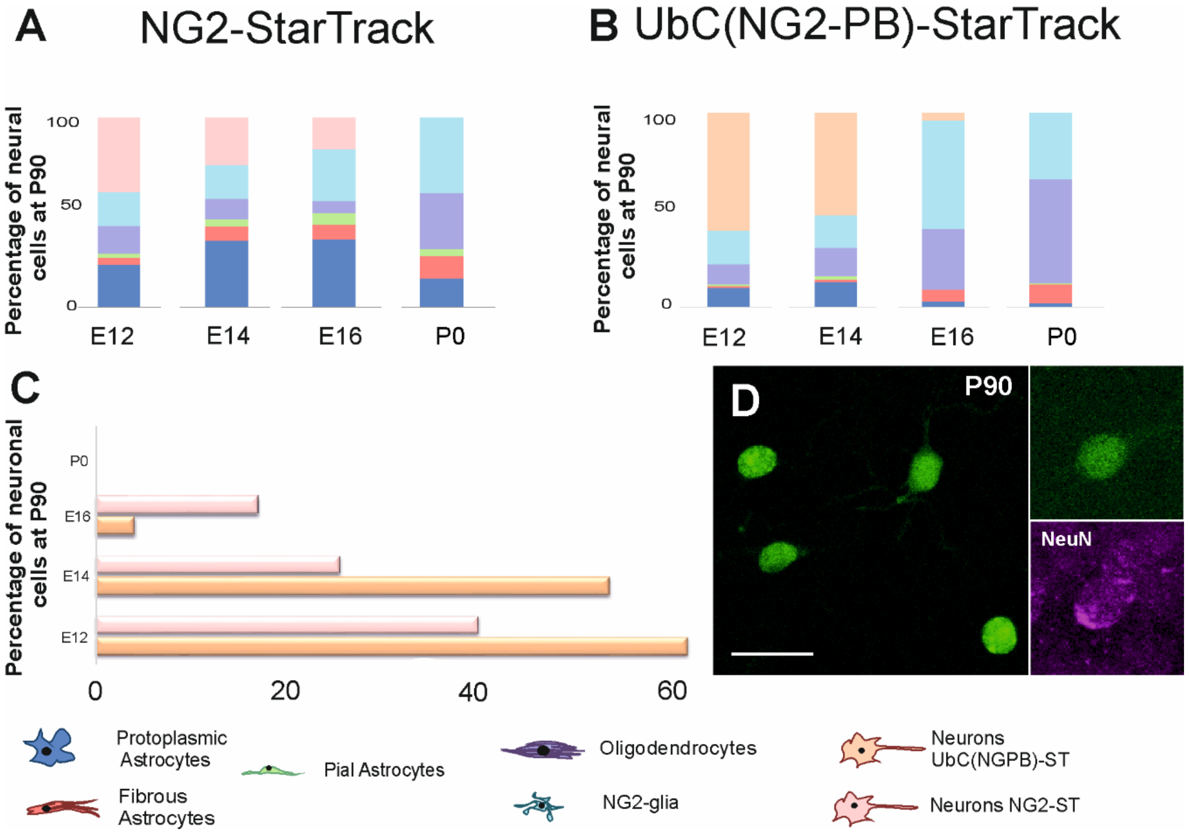

Figure S1. Cell progeny and lineage of NG2 progenitor cells. (A) Percentage of neural cell types at P90 after targeting progenitor cells at E12, E14, E16 and P0, using NG2-EGFP-StarTrack plasmids. **(B)** Percentage of neural cell types at P90 after targeting progenitor cells at E12, E14, E16 and P0, using UbC-(NG2-PB)-

EGFP-StarTrack. **(C)** Comparative graph showing the percentage of neuronal cells at P90 after targeting progenitor cells at E12 E14, E16 and P0, using either NG2-EGFP-StarTrack (soft pink) and UbC-(NG2-PB)-EGFP-StarTrack (soft orange) strategies. **(D)** P90 pyramidal neurons from dorsal cortex labelled with NG2-EGFP-StarTrack and coexpressed NeuN marker. Protoplasmic astrocytes are in blue, fibrous astrocytes in red, pial cells in green, oligodendrocytes in purple, NG2-cells in cyan. Neurons in soft pink and soft orange. Scale bar 50 μm.
